# Trends in and Factors Associated With Out-of-Pocket Spending for COVID-19 Hospitalizations From March 2020 to March 2021

**DOI:** 10.1001/jamanetworkopen.2021.48237

**Published:** 2022-02-14

**Authors:** Kao-Ping Chua, Rena M. Conti, Nora V. Becker

**Affiliations:** 1 Department of Pediatrics, Susan B. Meister Child Health Evaluation and Research Center, University of Michigan Medical School, Ann Arbor; 2 Department of Health Management and Policy, University of Michigan School of Public Health, Ann Arbor; 3 Department of Markets, Public Policy, and Law, Institute for Health System Innovation and Policy, Questrom School of Business, Boston University, Boston, Massachusetts; 4 Division of General Medicine, Department of Internal Medicine, University of Michigan Medical School, Ann Arbor

## Abstract

This cross-sectional study assessed the factors associated with out-of-pocket spending for COVID-19 hospitalizations from March 2020 to March 2021

## Introduction

During 2020, most private and Medicare Advantage insurers waived cost sharing for COVID-19 hospitalizations, thus protecting patients from financial liability.^1^ However, most insurers abandoned these cost-sharing waivers by August 2021.^2^ In an analysis of COVID-19 hospitalizations from March through September 2020, few privately insured or Medicare Advantage patients had cost sharing for hospital facility services, such as room-and-board charges, likely because waivers were widespread.^3^ We assessed whether the incidence of cost sharing for facility services increased in early 2021, when waivers began to expire.^4^ Among hospitalizations with patient cost sharing for facility services, we determined the factors associated with the magnitude of out-of-pocket spending for hospitalization care, thus identifying patients at elevated risk for large bills going forward.

## Methods

The IQVIA PharMetrics Plus for Academics database (IQVIA Inc) reports medical claims from 1.0 million patients covered by Medicare Advantage plans and 7.7 million patients covered by fully insured private plans across the US. Because the data were deidentified, the University of Michigan institutional review board exempted the analyses from review. This cross-sectional study followed the Strengthening the Reporting of Observational Studies in Epidemiology (STROBE) reporting guideline.

We included hospitalizations with a primary diagnosis of COVID-19 that began and ended between March 1, 2020, and March 30, 2021. We excluded hospitalizations if plans were the secondary insurer or if any hospitalization-associated claim had missing data for out-of-pocket spending or billing provider type. We calculated the monthly proportion of hospitalizations with cost sharing for facility services, a potential indicator that cost-sharing waivers were absent.^3^ Among these hospitalizations, we calculated the mean total out-of-pocket spending for all hospitalization-related services (see the eAppendix in the Supplement for details).

To identify factors associated with total out-of-pocket spending, we fitted generalized linear models with a log link and gamma variance function. Covariates were age group, sex, census region, admission month, plan type, intensive care use, and length of stay. To express coefficients as changes in out-of-pocket spending, we calculated average marginal effects.^5^ Analyses used Stata/MP, version 15.1 (StataCorp LLC) and 2-sided tests with α = .05.

## Results

Of 17 502 hospitalizations meeting inclusion criteria, 1052 (6.0%) were excluded, leaving 16 450 hospitalizations. These hospitalizations occurred among 15 625 patients. The mean (SD) patient age was 67.7 (14.0) years; 8109 patients (51.9%) were male.

For privately insured patients, the proportion of hospitalizations with cost sharing for facility services ranged from 2.2% to 8.8% between March 2020 and January 2021, then increased to 82.1% to 84.4% in February to March 2021. For Medicare Advantage patients, this proportion ranged from 0.3% to 2.7% between March 2020 and February 2021, then increased to 66.1% in March 2021 (Figure).^3^

**Figure.**
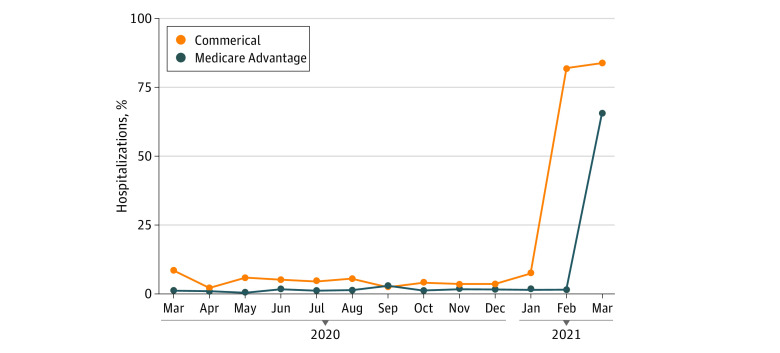
Proportion of COVID-19 Hospitalizations With Cost Sharing for Facility Services, From March 2020 to March 2021, PharMetrics Plus for Academics Facility services are those billed by hospitals, such as room-and-board charges. The ocurrence of cost sharing for these services may be an indicator that the hospitalization was covered by a plan that lacked a cost-sharing waiver for COVID-19 hospitalizations.^3^

Of 4926 hospitalizations for privately insured patients, 753 (15.3%) had cost sharing for facility services. Among these 753 hospitalizations, the mean total out-of-pocket spending was $3998 ($2698). Length of stay and non-Northeast residence were positively associated with this spending.

Of 11 524 hospitalizations for Medicare Advantage patients, 406 (3.5%) had cost sharing for facility services. Among these 406 hospitalizations, the mean total out-of-pocket spending was $1638 ($1062). Residence in the West and preferred provider organization enrollment were negatively associated with this spending, while length of stay was positively associated (Table).

**Table.** Characteristics of COVID-19 Hospitalizations in the Sample and Factors Associated With Total Out-of-Pocket Spending Among Hospitalizations With Cost Sharing for Facility Services, PharMetrics Plus for Academics^a^

Characteristic	Private insurance (n = 4926)			Medicare Advantage (n = 11 524)		
	Hospitalizations overall, No. (%)	Unadjusted total OOP spending among hospitalizations with cost sharing for facility services, mean (SD), $	Average marginal effect (95% CI)	Hospitalizations overall, No. (%)	Unadjusted total OOP spending among hospitalizations with cost sharing for facility services, mean (SD), $	Average marginal effect (95% CI)
Age group, y						
0-17	41 (0.8)	3942 (2452)	[Reference]	0	NH	NH
18-25	69 (1.4)	4148 (2817)	−23 (−2326 to 2281)	3 (0.0)	NH	NH
26-34	242 (4.9)	4505 (2837)	546 (−1501 to 2601)	19 (0.2)	1486 (NA)^b^	[Reference]
35-44	608 (12.3)	4353 (3346)	248 (−1693 to 2189)	71 (0.6)	1636 (215)	−192 (−454 to 70)
45-54	1390 (28.2)	3984 (2683)	−52 (−1953 to 1849)	344 (3.0)	1865 (934)	82 (−255 to 418)
55-64	2145 (43.5)	3915 (2465)	−188 (−2072 to 1697)	1228 (10.7)	1905 (1430)	175 (−289 to 639)
65-74	343 (7.0)	3352 (2206)	−684 (−2643 to 1274)	4027 (34.9)	1575 (951)	−52 (−331 to 227)
75-85	72 (1.5)	3707 (2781)	130 (−2488 to 2747)	3785 (32.8)	1549 (1090)	−38 (−381 to 306)
>85	15 (0.3)	188 (159)	−3667 (−5526 to −1808)	2047 (17.8)	1864 (1073)	2 (−331 to 336)
Sex						
Male	2896 (58.8)	4040 (2827)	[Reference]	5674 (49.2)	1707 (1209)	[Reference]
Female	2030 (41.2)	3910 (2496)	76 (−298 to 450)	5850 (50.8)	1579 (916)	16 (−149 to 181)
Region						
Northeast	596 (12.1)	3161 (2376)	[Reference]	2298 (19.9)	1684 (739)	[Reference]
Midwest	1447 (29.4)	4201 (2652)	1217 (652 to 1783)	5895 (51.2)	2086 (1242)	248 (−7 to 502)
South	2044 (41.5)	3996 (2528)	868 (375 to 1361)	2108 (18.3)	1669 (1089)	196 (−150 to 542)
West	814 (16.5)	4603 (3229)	1571 (905 to 2236)	1143 (9.9)	1032 (741)	−916 (−1151 to −681)
Plan type						
Health maintenance organization	1563 (31.7)	4050 (2675)	[Reference]	8907 (77.3)	1715 (1066)	[Reference]
Preferred provider organization	2429 (49.3)	3824 (2836)	110 (−452 to 673)	2473 (21.5)	1032 (810)	−640 (−876 to −405)
Consumer-directed health plan	934 (19.0)	4369 (2265)	−363 (−838 to 111)	0	NH	NH
Unknown	0	NH	NH	144 (1.2)	1552 (1246)	−299 (−1357 to 759)
Intensive care unit use^c^						
No	2880 (58.5)	4027 (2665)	[Reference]	6322 (54.9)	1541 (926)	[Reference]
Yes	2046 (41.5)	3916 (2762)	−342 (−750 to 66)	5202 (45.1)	1819 (1262)	9.5 (−197 to 216)
Month of admission^d^	NA	NA	79 (−12 to 169)	NA	NA	355 (95 to 615)
Month of admission squared	NA	NA	Omitted^e^	NA	NA	−20 (−32 to −8)
Length of stay	6.7 (7.1)^f^	NA	86 (34 to 138)	8.7 (8.4)^f^	NA	70 (46 to 95)
Length of stay squared	NA	NA	Omitted^e^	NA	NA	−0.9 (−1.3 to −0.6)

Abbreviations: NA, not applicable; NH, no hospitalizations with OOP spending for facility services in the subgroup; OOP, out-of-pocket.

^a^ Average marginal effects derive from an adjusted 1-part generalized linear model with a log link and gamma variance function, the latter of which was chosen based on the modified Park test.^6^ The sample included 17 502 hospitalizations meeting inclusion criteria, of which 882 were excluded because the plan was a secondary insurer and 167 were excluded because there was missing data for billing provider specialty or OOP spending on any hospitalization-associated claim, leaving 16 450 hospitalizations in the sample.

^b^ The SD could not be calculated because there was only 1 hospitalization with OOP spending for facility services in this subgroup.

^c^ Defined as the occurrence of 1 claim or more with a revenue code for intensive care unit or coronary care unit (code 0200-0209 or 0210-0219).

^d^ Coded as a continuous variable (eg, March 2020 = 1, April 2020 = 2, and March 2021 = 13).

^e^ Quadratic terms for month of admission and length of stay were only included if significant. For privately insured patients, these terms were not included in the regression, whereas they were in the regression for Medicare Advantage patients.

^f^ Refers to mean (SD) length of stay.

## Discussion

The proportion of COVID-19 hospitalizations with cost sharing for facility services surged in early 2021, when many insurers abandoned cost-sharing waivers.^4^ The mean out-of-pocket spending for these hospitalizations was $3998 for privately insured patients and $1638 for Medicare Advantage patients. This spending increased with length of stay and varied regionally.

Limitations include unclear generalizability to all private insurance and Medicare Advantage plans. Despite this limitation, findings suggest that patients could face substantial bills for COVID-19 hospitalization going forward. While the potential size of these bills may convince a few patients to become vaccinated, it could also prompt patients to delay seeking care irrespective of vaccination status, as both the vaccinated and unvaccinated are both now subject to cost sharing. Moreover, the threat of COVID-19 infection is not abating and may be increasing. Consequently, the widespread abandonment of insurer cost-sharing waivers is arguably premature.

## Notes

Supplement. **eAppendix.** Details on Methodology and Sample
