# Supplementary material for: Trends in and Factors Associated With Out-of-Pocket Spending for COVID-19 Hospitalizations From March 2020 to March 2021
Source: JAMA Netw Open. 2022 Feb 14;5(2):e2148237. doi: 10.1001/jamanetworkopen.2021.48237 (PMC8845007; doi:10.1001/jamanetworkopen.2021.48237)
Supplement: Supplement. — eAppendix. Details on Methodology and Sample [file jamanetwopen-e2148237-s001.pdf]

Supplemental Online Content

Chua KP, Conti RM, Becker NV. Trends in and factors associated with of out-of-pocket spending for COVID-19 hospitalizations from March 2020 to March 2021. *JAMA Netw Open*. 2022;5(2):e2148237. doi:10.1001/jamanetworkopen.2021.48237

eAppendix. Details on Methodology and Sample

This supplemental material has been provided by the authors to give readers additional information about their work.

© 2022 Chua KP et al. *JAMA Network Open.*

Appendix. Details on Methodology and Sample

Sample inclusion and exclusion criteria

We included hospitalizations that had a primary diagnosis of COVID-19 (ICD-10-CM diagnosis code: U071) and that began and ended between March 1, 2020 and March 30, 2021. Our claims database was considered to be complete through March 31, 2021 at the time we received the data at the end of September 2021 (six-month claims run-off). In our database, confinement episodes are defined as a contiguous string of facility room and board claims from the same billing identifier. The first and last dates of this string are the admission and discharge dates, respectively. If this string ends on March 30, 2021, we could be confident there was not another facility room and board claim on March 31, 2021, owing to the completeness of the data. However, if a string includes March 31, 2021, we cannot be sure that there were no other facility room and board claims on April 1, 2021. Thus, we required that the discharge date (last facility room and board claim) be on or before March 30, 2021.

Definition of facility and non-facility claims

When calculating "total out-of-pocket spending" among hospitalizations with cost-sharing for facility services, we summed out-of-pocket spending across all claims, including both facility and non-facility claims. These claims were defined similarly to our prior analysis (reference 3).

Facility claims were institutional claims with place of service code 21 (hospital) or 23 (emergency department) in which the "billing specialty" variable corresponded to a hospital. These included inpatient hospital services such as room and board, pharmacy, IV therapy, supplies, laboratory, radiology, blood bank, respiratory services, physical/occupational/speech therapy, and inpatient dialysis. These also included facility charges for emergency departments (e.g., revenue codes 0450-0459).

Non-facility claims included claims for professional and ancillary services submitted by clinicians and ancillary service providers, such as ambulance providers. Specifically, non-facility claims included one of the following 3 mutually exclusive types of claims:

1) Ambulance claims - place of service code 41-42 (ambulance) OR HCPCS codes between A0000-A9999

2) Clinician claims: (place of service 21 or 23) AND billing specialty variable = clinician

3) Miscellaneous claims: (place of service 21 or 23) AND billing specialty variable = other (including durable medical equipment providers and other facilities, such as dialysis centers)

© 2022 Chua KP et al. *JAMA Network Open.*
